# RETRACTED: Qian et al. Information System Model and Key Technologies of High-Definition Maps in Autonomous Driving Scenarios. *Sensors* 2024, *24*, 4115

**DOI:** 10.3390/s24247898

**Published:** 2024-12-11

**Authors:** Sensors Editorial Office

**Affiliations:** MDPI, Grosspeteranlage 5, 4052 Basel, Switzerland; sensors@mdpi.com

The Journal retracts the article titled “Information System Model and Key Technologies of High-Definition Maps in Autonomous Driving Scenarios” [1], cited above.

Following publication, concerns were brought to the attention of the publisher by one of the originally listed authors regarding the accuracy of the authorship list and the origins of this study [1].

Adhering to our standard procedure, an investigation was conducted by the Editorial Office and Editorial Board, which found that a number of the originally listed authors were unaware of this publication and did not consent to being listed as authors. Furthermore, following communications with the relevant institutions, the origins of the study could not be fully verified. As a consequence, the Editorial Board has lost confidence in the integrity of the findings and have decided to retract this publication [1] as per MDPI’s retraction policy (https://www.mdpi.com/ethics#_bookmark30, accessed on 5 December 2024).

This retraction was approved by the Editor-in-Chief of the *Sensors* journal.

All authors agree with this retraction.
